# Photo-Embossed Surface Relief Structures with Improved Aspect Ratios and Their Applications in Liquid Crystal Devices

**DOI:** 10.3390/polym14010171

**Published:** 2022-01-02

**Authors:** Xiulan Yang, Minzhao Gu, Qunmei Wei, Yang Zhang, Sihan Wu, Qin Wu, Xiaowen Hu, Wei Zhao, Guofu Zhou

**Affiliations:** 1 SCNU-TUE Joint Lab of Device Integrated Responsive Materials (DIRM), National Center for International Research on Green Optoelectronics, South China Normal University, No. 378, West Waihuan Road, Guangzhou Higher Education Mega Center, Guangzhou 510006, China; xiulanyang@yeah.net (X.Y.); minzhaogu@163.com (M.G.); qunmei.wei@m.scnu.edu.cn (Q.W.); yangzhang1114@126.com (Y.Z.); 2020023797@m.scnu.edu.cn (S.W.); xwhu@m.scnu.edu.cn (X.H.); guofu.zhou@m.scnu.edu.cn (G.Z.); 2 Guangdong Provincial Key Laboratory of Optical Information Materials and Technology & Institute of Electronic Paper Displays, South China Academy of Advanced Optoelectronics, South China Normal University, Guangzhou 510006, China; 3 Zhuhai Singyes New Materials Technology Co., Ltd., No. 9 Jinzhu Road, Technology Innovation Coast, High-Tech Development Zone, Jinding Town, Zhuhai 519000, China; wuqin@zhsye.com; 4 Shenzhen Guohua Optoelectronics Tech. Co., Ltd., Shenzhen 518110, China

**Keywords:** surface relief, photo-embossing, aspect ratio, liquid crystal device, polymer-free, light-regulating

## Abstract

Photo-embossing has been developed as a convenient and economical method for creating complex surface relief structures in polymer films. The pursuit for large aspect ratios of the photo-embossed structures has never stopped. Here, we demonstrate a simple strategy to obtain improved aspect ratios by adding a quick solvent developing step into the photo-embossing process. A good solvent for the monomer is used to remove unreacted monomers from the unexposed region, resulting in deepened valleys of the surface reliefs. In a polymer film as thin as 2.5 µm, the height of the surface reliefs can be increased by a factor of three to around 1.0 µm. This strategy is also shown to be compatible with other methods used to improve the aspect ratios of the photo-embossed structures. Lastly, we employ these surface relief structures in the fabrication of liquid crystal (LC) devices and investigate their performances for visible light regulation.

## 1. Introduction

Liquid crystals (LCs), as a classic soft material, demonstrate a rich variety of intriguing optical, electrical, and magnetic properties, as well as stimuli-responsiveness, making them ideal candidates for the next-generation smart materials and devices [1,2,3,4]. Owing to their anisotropic molecular shape, LC materials are highly sensitive to surface chemistry, geometric confinement, surface topography, and external stimuli such as heat, light, and electric or magnetic fields [5,6,7,8,9]. Among these factors, surface topography has been broadly used as a simple tool to produce uniform or patterned alignment in nematic LCs, and to control the molecular alignment and topological defects in smectic LCs or LC polymers [6,10,11,12,13]. For example, rubbing a velvet cloth on a substrate coated with polyimide (PI), polyvinyl alcohol (PVA), or other polymers often generates one-dimensional (1D) microgrooves on the polymer surface, leading to the homogeneous alignment of nematic LCs [14].

To accommodate the need for fabricating surface topographical structures ranging from the nano- to the micro-scale, abundant techniques have developed [12,15,16,17,18,19,20,21]. Yi et al. took micro-scale groove patterns prepared with nanoimprint lithographically (NIL) and achieved successful alignment of nematic LCs [15]. Lee et al. later constructed nano-scale 1D patterns using NIL and obtained a similar alignment effect [16]. Ion-beam (IB) irradiation has also been employed to change both the topographical and chemical characteristics of the polymer surface and induce unidirectional LC alignment [17,18]. Another convenient method is photolithography, which is often combined with soft lithography to produce complex surface topographies and realize control over the local alignment of the LCs [10,12,22]. Two photon polymerization (TPP) is also used to fabricate multiple surface-relief structures and attain uniform or space-variant LC alignment [20]. Other than aligning the LCs, random surface topography is also studied for its effect on enhancing the haze of a light-regulating smart window based on the polymer-stabilized liquid crystals (PSLCs) [23]. Azobenzene based LC polymer has been demonstrated to form surface relief gratings after exposure to two-laser beam interference as well [24,25]. Despite the great number of successful examples, research to develop surface modification methods for the fabrication of novel LC devices is still progressing.

On the other hand, photo-embossing has been extensively explored as an economical method to produce complex surface-relief structures [26,27,28,29,30,31,32,33,34,35,36]. The photo-embossing method offers several benefits: it relies on the polymerization-induced diffusion mechanism which is flexible to adjustments; it works with a wide range of systems, including typical photo-polymerizable (meth)acrylates, supramolecular polymers, organic semiconductors, and fluoropolymers; and it can be applied to not only polymer thin films but also to polymer fibers. One limitation of the method is that the aspect ratio of the photo-embossed structures is usually low (within a range of 0.05 to 0.2) [28]. When the initial film thickness is limited to a few microns, this value is only around 0.05 [26]. Here, aspect ratio is defined as the quotient of the height and the width of a structure. Since the low aspect ratio is limiting the application potential of the method, many attempts have been made to improve the value [27,28,29,31]. Nevertheless, the examples of using the photo-embossing method in LC-related systems and applications remain scarce.

Here, we present a simple yet effective method to increase the aspect ratio of the photo-embossed structures in a thin polymer film. By introducing a quick solvent developing step, the aspect ratio can be improved by a factor of 3. This factor could be further enlarged if combined with other strategies such as using inhibitors or chain transfer agents in the free radical polymerization. This method is shown to work well for both the 1D line pattern and the 2D square and checkerboard patterns. Subsequently, the photo-embossed structures are explored in light-regulating LC devices based on a polymer-free design. The devices demonstrate good potential for controlling the transmitting and scattering of incident light.

## 2. Experimental

### 2.1. Materials

**Figure 1:**
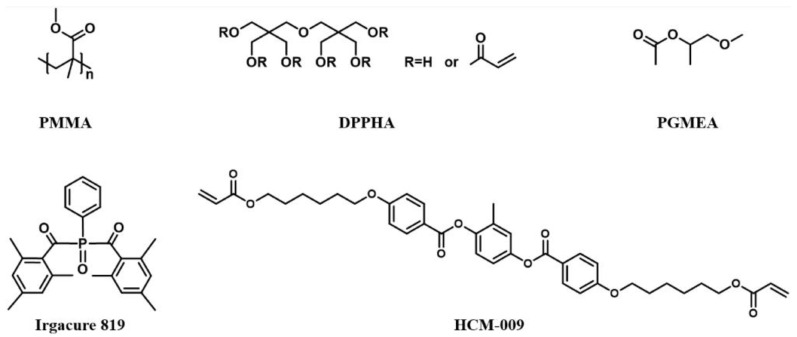
Chemical structures of the materials used in the study.

The blend used for preparing photo-embossed surface relief structure contains 45.5 wt.% polymethyl methacrylate (PMMA, Sigma Aldrich, Darmstadt, Germany) as the polymer matrix, 45.5 wt.% dipentaerythritol penta-/hexa-acrylate (DPPHA, Sigma Aldrich, Darmstadt, Germany) as the reactive monomer, and 9.0 wt.% Irgacure 819 (TCI Development Co., Ltd., Shanghai, China) as the photo-initiator (Figure 1). Propylene glycol monomethyl ether acetate (PGMEA, Sigma Aldrich, Darmstadt, Germany) was used as the solvent to prepare the solution for spin-coating.

LC of negative dielectric anisotropy (HNG30400-200, T_NI_ = 94 °C, Δε = −8.3, Δ*n* = 0.149) was obtained from Jiangsu Hecheng Display Technology Co., Ltd. (Nanjing, China) and used for all the devices. The PSLC device was prepared following our previous report [37], using the LC monomer HCM-009 (Figure 1).

### 2.2. Preparation of Photo-Embossed Structures

**Figure 2:**
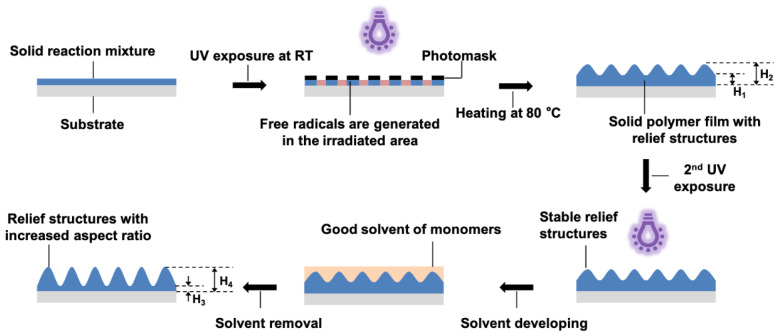
Schematic representation of the photo-embossing process, including the solvent developing step. RT stands for room temperature.

The surface relief structures are prepared on the ITO-coated glass slides, which were treated by UV-ozone (BZS250GF-TC, Shenzhen HWOTECH Co., Ltd., Shenzhen, China) for 20 min before use. The preparation of the photo-embossed structures largely follows the previous report, and is schematically shown in Figure 2. The slight modifications are the 2nd UV exposure without photomask (25 mW/cm^2^, 5 min) and the additional solvent developing step, in which a good solvent for the monomer was used and spin-coating (3000 rpm, 30 s), was performed for solvent removal. The UV exposure was conducted on a lithography machine (URE-2000s, Institute of Optics and Electronics, Chinese Academy of Sciences, Chengdu, Sichuan, China). The final UV flood exposure was performed using a UV curing machine (SFMY-130130U, Guangzhou Bangwo Electronic Technology Co., Ltd., Guangzhou, China).

### 2.3. Characterization

Surface profilometry system Leica DCM8 (Leica, Wetzlar, Germany) was used to measure the height and topography of the photo-embossed surface relief structures. The transmission spectra of the devices were obtained using an Ocean Optics Maya 2000Pro spectrophotometer (Ocean Optics, Dunedin, FL, USA) using ITO glass as the background. Polarized optical microscopy (POM) was performed using a Leica DM 2700P (Leica, Wetzlar, Germany).

## 3. Results and Discussion

### 3.1. Photo-Embossed Surface Relief Structures with Improved Aspect Ratios

The photo-embossing method has been systematically optimized since the first time it was reported [38,39]. The typical process includes four main steps: (1) preparation of a thin polymer film that contains the polymer binder, the reactive monomers, and the photoinitiator; (2) UV exposure through a photomask at room temperature (RT) during which free radicals are generated but confined within the irradiated areas; (3) heating of the sample which enables monomer diffusion from the unexposed to the irradiated areas, which leads to the formation of the surface relief structures; and (4) flood UV exposure at RT to ensure complete consumption of the monomers and photoinitiators and fixation of the formed relief structures [26]. Although different techniques have been employed to tune the kinetics of the photopolymerization, the conversion of monomers cannot be completed before the final flood exposure step. Importantly, free radical polymerization is known to be influenced by the presence of air, and even when used in a nitrogen atmosphere the lifetimes of the free radicals are still limited. In addition, the diffusion of monomers is limited in the viscous polymer matrix. The reacted monomers near the boundary of the exposed and unexposed areas will increase the matrix viscosity and impede further diffusion of monomers. For such reasons, there should be unreacted monomers left in the unexposed area after the initial formation of the relief structures. Removing these monomers without affecting the other parts should in theory enlarge the aspect ratio of the formed structure.

**Figure 3:**
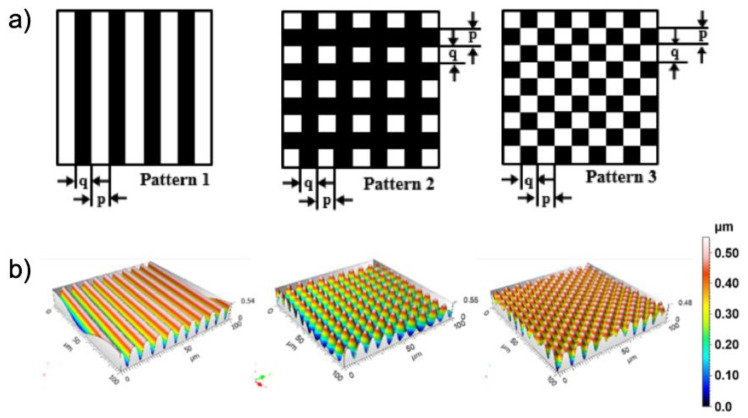
(**a**) Schematic of three pattern masks. The black part represents the masked area where the UV light is blocked. The period of photo-embossed structure is defined as p + q, where p = q in the current study; (**b**) the corresponding three-dimensional topographies of the photo-embossed surface reliefs.

In order to verify the idea, three photomask patterns are used to fabricate the surface relief structures. These are a 1D line pattern, and 2D square and checkerboard patterns. Following the reported procedures, regular surface structures can be successfully obtained after photo-embossing (Figure 3). Simply by adjusting the size (*p*, *q*) of the photomask patterns, the size of the formed structures will change accordingly. It is known that the monomer and the corresponding polymer may have different solubility in a given solvent, especially when the polymer is crosslinked. Selective removal of the unreacted monomers is thus possible by choosing a proper good solvent, which is N-methylpyrrolidone (NMP) in our case. We added a 2nd UV exposure step to stabilize the formed relief structures. During the solvent developing step, NMP is introduced on top of the surface relief structures and then spun off after a few seconds. Nitrogen is used to blow off the residue NMP before the final flood UV exposure is conducted. The line pattern with a 10 µm period is examined first. It is found that the aspect ratio of the formed structure doubles after the solvent developing process (Table 1, sample A). In contrast, if a non-solvent is used, this process does not contribute to any increase in the aspect ratio (Table 1, sample B). Detailed analysis reveals that in sample A the distance from the peaks to the substrate (H_2_, H_4_) remains unchanged, while the distance from the valley to the substrate (H_1_, H_3_) decreases, supporting the previous hypothesis of removing unreacted monomers from the unexposed area (i.e., the valley). None of these two distances change after the process in sample B, which is reasonable given the non-solvent nature.

**Figure A1:**
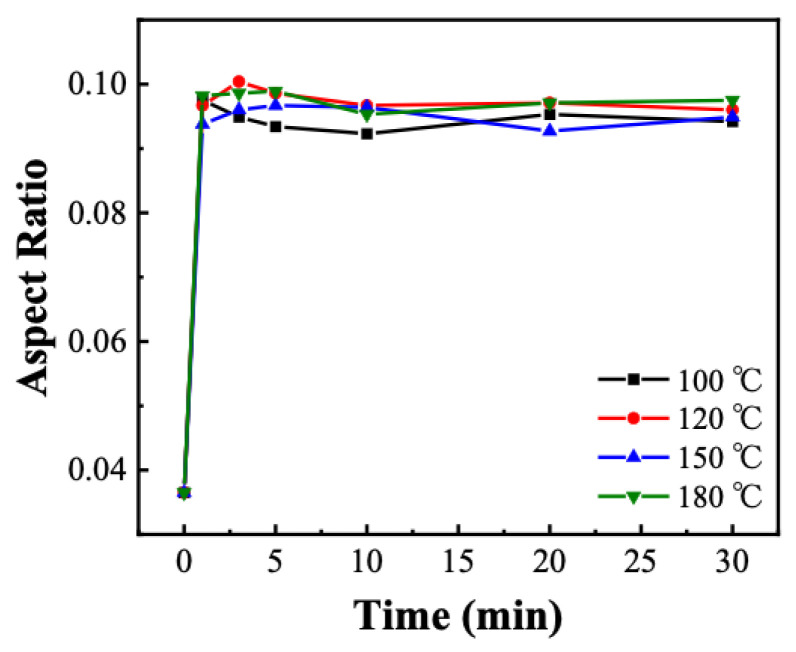
The effect of heating temperature and time in the solvent developing process on the final aspect ratio of the photo-embossed structures.

The solvent developing process was optimized to obtain even higher aspect ratios. A heating step is first added to the process after N_2_ purge, which was found to give a slight increase in the aspect ratio (Table 1, sample C). This is presumably due to the remaining active free radicals following the heating step in the typical photo-embossing process. The added NMP solvent may facilitate diffusing of unreacted monomers into the exposed areas, resulting further growth of the surface relief structures. Increasing the heating temperature or extending the heating time do not show any further improvement (Figure A1 (Appendix A)). In addition, a solvent soaking step of 1 min is used. In contrast, in the spin-coating process the solvent or solution mixture is placed on top of a substrate and immediately followed by spinning of the substrate. The soaking step is expected to promote dissolving of the unreacted monomers, and therefore ‘etching’ further into the valleys. In such way, the aspect ratio of sample D can reach 0.1, which is more than three times higher than the control sample Z. This solvent developing strategy can be applied to the other photo-embossed patterns to obtain improved aspect ratios as well (Table A1).

**Figure 4:**
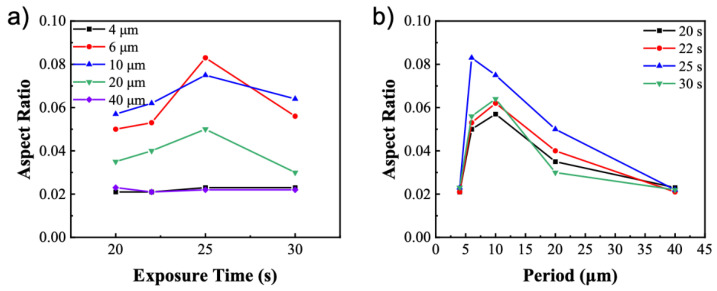
Aspect ratio of the photo-embossed structures as a function of (**a**) UV exposure time for different periods and (**b**) period of photomask for different exposure times.

**Figure A2:**
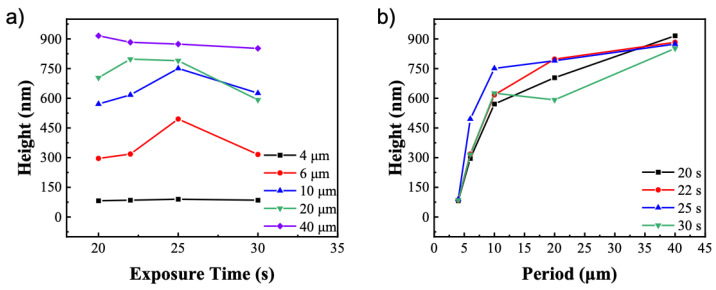
Height (distance to the substrate) of the photo-embossed structures as a function of (**a**) UV exposure time for different periods and (**b**) period of photomask for different exposure times.

In addition, the conditions for photo-embossing are investigated to understand their impact on the solvent developing process, in particular the exposure time and the period of line patterns. It has been shown that there exists an optimum energy dose due to the competing kinetics of diffusion and polymerization [26]. Monomer diffusion is strongly hindered at overexposed conditions, while monomer conversion is low in the polymerization at sub-optimal exposure conditions. In the current study, the UV intensity is fixed at 25 mW/cm^2^ while the exposure time is extended from 20 to 30 s. A similar optimum energy dose of 750 mJ/cm^2^ has been observed for every period (Figure 4a). This value agrees well with the previous study, which can also be used to explain the observed optimum [26]. When the aspect ratios are plotted against the periods of photomasks, it clearly demonstrates that there is an optimum period for every energy dose. Since aspect ratio is calculated as the quotient of the height and width of a structure, larger periods will naturally lead to smaller quotient values. In contrary, at sub-optimal periods, the fraction of unreacted monomers in the valley regions may be small due to short diffusion lengths and limited number of materials, resulting in low structure heights after solvent developing (Figure A2).

One thing to note is that the aspect ratio obtained in our experiment is very similar to the reported result by Sánchez et al. [26]. In the previous report, for a line pattern of 10 µm period and initial film thickness of around 9 µm, the final relief structure has a height between 300 and 400 nm. The final height is shown to increase gradually with increasing film thickness. It is reasonable to obtain an aspect ratio of 0.03 for the control sample Z since the initial thickness is only around 2.5 µm. Therefore, the increase in aspect ratio after introducing the solvent developing strategy is quite remarkable given how thin the initial polymer film is. In fact, if combined with an initial film of larger thickness, or the addition of a free radical polymerization inhibitor, the solvent developing strategy continues to be effective in improving the aspect ratio of the photo-embossed structures (Table A2). The reason the current study chooses such a thin film is the fact that the target applications require the fabrication of LC cells and spin-coating is used to produce the initial film of uniform thickness.

### 3.2. Liquid Crystal Devices Based on Photo-Embossed Surface Relief Structures

With the photo-embossed surface relief structures successfully made, we move to study their application in LC-based light-regulating devices. A polymer-free design is chosen for the study. The elimination of the polymer network structure is expected to avoid the stress relaxation of the network and thus the deterioration of device performance. Homeotropic alignment using a polyimide (PI) layer is used in the design. The solvent of the PI precursor solution is chosen to be NMP, so the spin-coating of such a solution to prepare the PI layer and the solvent developing process can be conveniently combined. Based on the previous results, photomasks with 10 µm period are used for the study.

**Figure 5:**
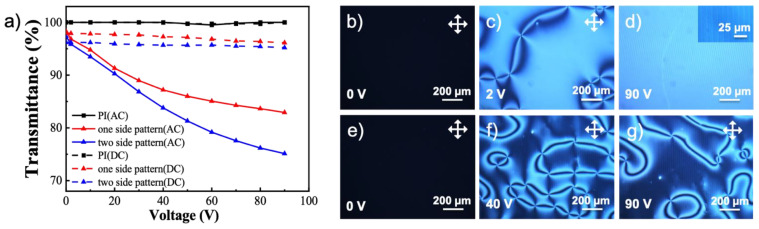
(**a**) The transmittance of three types of liquid crystal devices after applying DC and AC electric fields. The first type of device has no photo-embossed structure. The second type has photo-embossed structure of the line pattern on one side, while the third type on both sides. The POM images of the third type device under AC and DC fields of different voltages are shown in (**b**–**g**). (**b**), (**c**) and (**d**) correspond to AC field of 0 V, 2 V, 90 V, respectively. (**e**), (**f**), and (**g**) correspond to DC field of 0 V, 40 V, 90 V, respectively.

**Figure A3:**
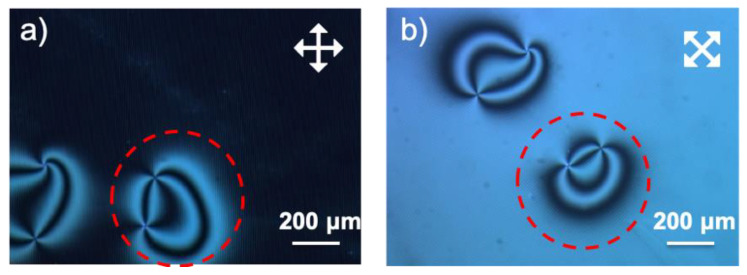
AC field will promote the oscillating motion of LC molecules that lead to alignment along the line shaped relief structures. The same LC cell viewed under POM (**a**) without rotating and (**b**) rotated 45°.

The influence of cell design is first examined along with the driving scheme. The line pattern is used for making the surface reliefs and the cell gap is set to be 10 µm. An LC cell made by flat ITO substrates coated with PI alignment layer is used as the control. The control sample shows almost no transmittance change no matter if a DC or an AC field is applied. In contrast, the LC cells with the surface relief structures do show tunable transmittance. In terms of the cell design, when only one substrate has the photo-embossed structures, the transmittance drop is larger than device with structures on both sides (Figure 5a). Here the lines lie parallel on both substrates. In addition, the use of a DC field is compared to an AC field to drive the devices, but always shows less change in transmittance. The LC cell composed of two substrates coated with the photo-embossed structures is further inspected using POM. Despite the undulated surface relief structures, the device presents perfect homeotropic alignment without any field applied (Figure 5b,e). The morphology turns into a typical nematic Schlieren texture with either AC or DC field (Figure 5c,f). However, as the voltage further increases to 90 V, the sample under DC field still contains a significant number of defects (Figure 5g), whereas the AC field has erased nearly all the defects and the LC molecules align homogeneously (Figure 5d). This result is likely due to the oscillating motion of the LC molecules under an AC field will promote rotation and alignment in the valley region [15] (Figure A3), while the homeotropic aligning effect dominates near the peaks of surface reliefs. An alternating homeotropic-homogeneous aligned line texture is therefore formed, giving a pattern of alternating dark-bright lines (Figure 5d, insert). The transmittance drop is possibly due to the scattering of incident light caused by such grating-like morphology. Moreover, when the photo-embossed relief structures are used on both sides of the LC cell, the scattering effect is stronger than the sample with relief structure only on one side, and thus causes larger drop in the transmittance.

**Figure 6:**
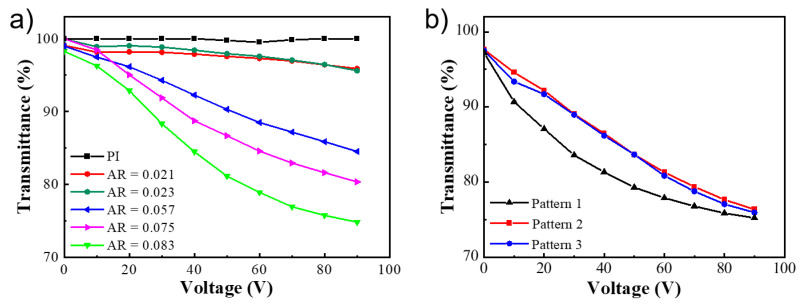
The transmittance as a function of AC voltage for polymer-free liquid crystal devices based on photo-embossed structure, with (**a**) increasing aspect ratios of pattern 1, and (**b**) three different patterns.

Next, we move to investigate the influence of aspect ratio and pattern of the photo-embossed structures. The LC cell is composed of two substrates coated with the same structures, and an AC field is used to drive the device. The cell gap is kept at 10 µm. Samples with increasing aspect ratios of undulated lines are first examined, with a PI alignment layer used as the control again (Figure 6a). As the aspect ratio increases, the transmittance drop becomes monotonically bigger, with the highest value being around 25% at 90 V. When observed under POM, these samples present similar changes from a perfect homeotropic alignment to a typical nematic Schlieren texture, and finally to an alternating homeotropic-homogeneous aligned line texture. With the aspect ratio becoming bigger the microscale 1D grooves will presumably provide an increasingly strong homogeneous aligning effect in the valley region towards the LC molecules. Consequently, the resultant grating-like morphology has a more pronounced refractive index mismatch, and the scattering of incident light is stronger. The influence of the photo-embossed pattern has also been compared (Figure 6b). It is interesting that all three patterns lead to a similar transmittance drop at 90 V, likely since the rotation and alignment of the LC molecules have reached a stable state at this voltage. Since all three patterns have similar heights for the photo-embossed structures (Table A3), the scattering of light may be similar and thus the transmittance drops. The slightly different T-V curve shapes might be due to the surface pattern and the resulting anchoring strength difference on the LC molecules.

## 4. Conclusions

In summary, we have successfully developed a new strategy to improve the aspect ratios of surface relief structures obtained via photo-embossing. After the monomer diffusion and formation of the surface relief structures due to photo-polymerization, an additional solvent developing step is introduced. A good solvent for the monomer is used to dissolve and remove the unreacted monomers from the unexposed regions. In contrast, the monomers are largely consumed to form a crosslinked network that cannot be removed in the exposed region. As a result, the valleys of the surface reliefs will be deepened after the solvent developing step, leading to increased aspect ratios. This strategy is simple and effective, and can increase the aspect ratio by a factor of three in a relatively thin polymer film of only 2.5 µm. More importantly, it is demonstrated to be compatible with other methods, such as increasing film thickness and adding free radical polymerization inhibitors, if further increase of the aspect ratio is needed. Lastly, we have explored the application of these photo-embossed surface relief structures in LC devices targeting visible light regulation, based on a polymer-free design. The transmittance drops around 25% with an AC field of 90 V applied, likely due to the scattering effect of the regular photo-embossed relief structures. Given the simple and economical nature of the photo-embossing method, it is expected to facilitate other applications in LC devices as well.

## Figures and Tables

**Table 1:** Structure sizes measured for the four samples treated with different solvent developing conditions, and a control sample without any solvent treatment. The line pattern with 10 µm period is used for all the samples.

Sample ID	Experimental Conditions	H_1_/H_2_	Aspect Ratio	H_3_/H_4_	New Aspect Ratio
Z (control)	No solvent developing step	2.3/2.6	0.03	--	--
A	Spin-coat with NMP Purge with N_2_	2.3/2.6	0.03	1.9/2.5	0.06
B	Spin-coat with water Purge with N_2_	2.3/2.6	0.03	2.3/2.6	0.03
C	Spin-coat with NMP Purge with N_2_ Heat at 120 °C for 3 min	2.4/2.7	0.03	1.8/2.6	0.08
D	Soak with NMP for 1 min Spin off NMP Purge with N_2_ Heat at 120 °C for 3 min	2.3/2.6	0.03	1.5/2.5	0.10

## Data Availability

Not applicable.

## Appendix A

**Table A1:** Structure sizes measured for three samples with line pattern (#1), square pattern (#2), and checkboard pattern (#3), respectively. The periods are set to be 10 µm for all three samples.

Pattern	H_1_/H_2_	Aspect Ratio	H_3_/H_4_	New Aspect Ratio
#1	2.3/2.6	0.03	1.5/2.5	0.10
#2	3.0/3.3	0.03	2.8/3.4	0.06
#3	3.2/3.4	0.02	2.9/3.4	0.05

**Table A2:** Structure sizes measured for samples with increased initial film thickness (sample E) and radical polymerization inhibitor t-butyl hydroquinone (TBHQ) in the film (sample F), as compared to sample D. The periods are set to be 10 µm for all three samples.

Sample ID	H_1_/H_2_	Aspect Ratio	H_3_/H_4_	New Aspect Ratio
D	2.3/2.6	0.03	1.5/2.5	0.10
E	4.5/4.9	0.04	3.7/4.8	0.11
F	2.2/2.7	0.05	1.3/2.6	0.13

**Table A3:** Structure height of the surface reliefs for line pattern (#1), square pattern (#2), and checkboard pattern (#3) used for LC cells in the polymer-free design.

Pattern	Structure Height before Coating of PI (nm)	Structure Height after Coating of PI (nm)
#1	299 ± 8	552 ± 48
#2	200 ± 6	524 ± 12
#3	321 ± 18	523 ± 13
